# Fully Noncontact Hybrid NDT for 3D Defect Reconstruction Using SAFT Algorithm and 2D Apodization Window

**DOI:** 10.3390/s19092138

**Published:** 2019-05-08

**Authors:** Hossam Selim, José Trull, Miguel Delgado Prieto, Rubén Picó, Luis Romeral, Crina Cojocaru

**Affiliations:** 1 Physics Department, Universitat Politècnica de Catalunya, Rambla Sant Nebridi 22, 08222 Terrassa, Barcelona, Spain; hossam.eldin.mohamed.selim@upc.edu (H.S.); jose.francisco.trull@upc.edu (J.T.); crina.maria.cojocaru@upc.edu (C.C.); 2 Electronic Engineering Department, Universitat Politècnica de Catalunya, Rambla Sant Nebridi 22, 08222 Terrassa, Barcelona, Spain; miguel.delgado@upc.edu (M.D.P.); luis.romeral@upc.edu (L.R.); 3 Instituto de Investigación para la Gestión Integrada de Zonas Costeras, Universitat Politècnica de València, Paranimf 1, Grao de Gandia, 46730 València, Spain

**Keywords:** laser ultrasonics, noncontact transducers, defects, NDT, SAFT, synthetic aperture, apodization, weighting function, 3D reconstruction

## Abstract

Nondestructive testing of metallic objects that may contain embedded defects of different sizes is an important application in many industrial branches for quality control. Most of these techniques allow defect detection and its approximate localization, but few methods give enough information for its 3D reconstruction. Here we present a hybrid laser–transducer system that combines remote, laser-generated ultrasound excitation and noncontact ultrasonic transducer detection. This fully noncontact method allows access to scan areas on different object’s faces and defect details from different angles/perspectives. This hybrid system can analyze the object’s volume data and allows a 3D reconstruction image of the embedded defects. As a novelty for signal processing improvement, we use a 2D apodization window filtering technique, applied along with the synthetic aperture focusing algorithm, to remove the undesired effects due to side lobes and wide-angle reflections of propagating ultrasound waves, thus enhancing the resulting 3D image of the defect. Finally, we provide both qualitative and quantitative volumetric results that yield valuable information about defect location and size.

## 1. Introduction

Nondestructive testing (NDT) techniques are widely used for embedded crack detection inside solid materials, with effective results in the quality control and material inspection strategies in modern industry [1]. Ultrasound can propagate in solid media as longitudinal waves (parallel to direction of propagation), shear waves (perpendicular to direction of propagation), surface waves (Rayleigh waves that travel in thick materials at a depth of one wavelength), and plate waves (surface waves that propagate only in thin materials a few wavelengths in thickness). Longitudinal and shear waves are the two modes of propagation most widely used in NDT applications in homogenous materials. Any specific defect has to be larger than half the wavelength of the propagating ultrasound wave. Otherwise, it will not have any influence of the wave propagation [2,3,4]. Ultrasound transducers are considered the most common devices for NDT inspection thanks to their wide frequency band selection, small sizes, and ability to work in harsh environments [5,6]. Ultrasound transducers can be used in exciter or receiver modes, or pulse-echo or pitch-catch modes, respectively [7,8]. Either of these two modes is favored to be used for NDT applications depending on the typical application and availability of transducers. However, the use of ultrasonic transducers both as exciters as well as receivers of ultrasound has always faced some limitations, including the difficulty to use them in remote areas, where it is difficult to place the transducer in contact with the object under inspection [9]. The limited resolution and weak generated power of noncontact transducers used as exciters lead to a reduction of the ultrasound waves’ penetration depth, limiting their application mostly to the detection of near-surface defects. Noncontact conventional transducers are, however, suitable as receivers. Typical NDT techniques use contact transducers fixed in a single position on the object’s surface. Although in contact mode the received signal has a higher signal to noise ratio (SNR) with higher gain and less attenuation, the mode has an important drawback when the same detector has to be used to scan a certain area; when the detector is moved from one point to another, the coupling factor changes, rendering impossible a quantitative comparison of the two measurements. Moreover, automatic scanning is not possible to implement with contact sensors. On the contrary, a noncontact transducer can be programmed to automatically scan a larger area, with the drawback of signal attenuation due to the airgap and the weaker sensitivity to detect small variations in the ultrasound signal.

In the last few years advanced sensor designs have been of interest to improve damage detection. For instance, they have been used in advanced nonlinear technologies applied to the design of ultrasonic transducer using phononic crystals (PCs). PCs filter out unnecessary second harmonic frequencies and enhances spatial focusing properties enabling detection of nonlinear effects without modifying the acquired signals, and allowing one to determine the spatial location of the damage [10,11].

Signal processing usually implemented through contact or noncontact ultrasonic techniques is based on the extraction of the time-of-flight (TOF) corresponding to the reflected echoes generated by the embedded defects. Several algorithms have been proposed, including the B-scan [12], Fourier-transform, short-time Fourier-transform (STFT) [13], wavelet transform (WT) [14,15], time reversal [16], and synthetic aperture focusing technique (SAFT) algorithms [12,17,18,19,20]. Each algorithm has advantages and limitations. For instance, the B-scan algorithm can give time domain and 1D space domain information of the defect by combining multiple A-scan measurements in cascade [18]. The Fourier-transform has the ability to detect the frequency response of the detected signal without giving detailed information about the corresponding TOF of a certain frequency component. STFT and WT can detect information about both frequency and TOF with a certain level of uncertainty. However, WT is more flexible and accurate than STFT due to the fact that the window size in the WT changes with frequency and time, whereas the window size, in the case of STFT, is fixed, which limits the resolution of the algorithm [21]. Moreover, time reversal techniques rely on the principle of detecting the ultrasound wave field using a receiver and transmitting the same wavefield to the source reversed in time, producing a convergence of the signal towards the initial source position. If the source of this signal is a defect, then the convergence of the reversed signal will occur at the defect position allowing its visualization [16]. Finally, the SAFT technique relies on the principle of delay and sum (DAS) that generates a focused image of the defect out of multiple unfocused images. This focused image has much higher amplitude at the defect position compared with healthy positions inside the object of interest. The SAFT has the advantage of being able to visualize the whole volume providing 2D or 3D information about the object depending on the resolution and the number of scans performed on the object [22]. In a recent work, SAFT is used with ultrasonic transducers to generate an image of the embedded defects in the sample under test representing a viable solution for signal processing [17,19,23]. An apodization function can be enclosed in the SAFT algorithm in order to enhance the reconstructed image by removing the effect of side lobes. The function can be used in 1D when one performs a line scanning, as described in the literature. Using this approach, a window function is selected to cover the size of the synthetic aperture to filter out the signals detected by the receiving transducer at angles greater than the beamwidth angle. This signal conditioning removes the effect of side and redundant echoes that are not generated by the main lobe of the incident signal. The beamwidth angle depends on the frequency passband, the size of the transducer and the wave velocity. When the scanning is performed in 2D (i.e., a scan area), the apodization window is extended from a line to an area since the beamwidth angle is extended to become a solid angle [24,25]. The size of the synthetic aperture changes with the depth of the scattering point. Therefore, the size of the apodization function should be adjusted accordingly to cover the synthetic aperture area. A drawback of apodization is the possible change of the main lobe width, which affects the lateral resolution [26,27]. Hence, good selection of the apodization function and its width help optimize results with minimum lateral resolution deficiency. Rectangular and Hanning apodization functions are commonly used in the SAFT analysis [24].

Advanced signal processing algorithms are also used to detect defects by analyzing echoes received by guided waves that travel long distances without substantial attenuation in irregular-shaped waveguides. Any perturbation in the detected signal represents a defect. Signal processing is applied by exploiting dispersion compensation properties of a warped frequency transform (WFT) [28]. In addition, other techniques using nonlinear contact dynamics have shown interesting results for detecting defects inside materials using resonance and non-resonance vibrations [29]. Nonlinear elastic wave spectroscopy combined with the elastic energy localization of the time reversal mirror can isolate surfacial nonlinear scatterers in solids [30]. Combining Time reversal and laser vibrometry made it possible to locate an impact event by applying a correlation of the measured impact response and a set of measured test data acquired at various grid points along the specimen surface [31].

As an alternative to ultrasonic methods, all-optical systems for ultrasound generation and detection have emerged. Laser generated ultrasound (LGU) technique is used for excitation, while optical interferometry setups are used for signal detection. These all-optical methods have high resolution and the ability to send and receive signals remotely at intensities that are not achievable by conventional transducers [32,33]. This implies larger penetration of the ultrasound to deeper areas inside the object and the ability to detect deeper embedded defects. The main drawback of interferometric optical detection, apart from the fact that it is expensive, is the stability criterion. The system needs to be isolated against surrounding vibrations, making it difficult to apply in practical industrial applications [32]. Due to these drawbacks, some of all-optical NDT methods can detect defect sizes at a micrometer scale. However, there are some exceptions since amongst optical NDT methods there is also, for example, infrared thermography, which is not affected by the previously mentioned drawbacks [34].

In this work, we propose a hybrid system that combines laser-induced ultrasound as an exciter, and a conventional noncontact transducer as a receiver. This method combines the advantages of both technologies to obtain a full, contactless, 3D reconstruction of a defect with good resolution, and to improve the applicability of the system in industrial applications [35]. The target object inspected is a metallic cube made of cast aluminium with an embedded cylindrical glass defect that we detect using the SAFT algorithm. In the proposed method, both the exciter (the laser spot) and the receiver (only one noncontact transducer) are programmed to automatically scan 2D surfaces of three different faces of the object, which emphasizes the 3D reconstruction of the defect at different perspectives/angles. The recorded ultrasound signals are processed and superimposed using the standard SAFT algorithm for 3D defect reconstruction. The results of SAFT at each face gives information about the defect from one view angle. Superimposing the results of the three faces together is then performed to provide information about the defect from different angles/perspectives. We apply the three faces scanning and SAFT algorithm processing as a contribution to expand the one face scanning for SAFT analysis that is described in the literature [12,17,18,19,20,22,23]. In addition to the SAFT method, an enhanced 2D apodization function is implemented as a novel contribution that eliminates the side lobes generated by the exciter as an artifact in the input signal [24]. This technique results in improved quality of the reconstructed image with less redundant or unnecessary shadows. To our knowledge, the apodization function for signal processing using the SAFT algorithm was previously applied to 1D geometries for 2D defect reconstruction. Here, we have developed the extended 2D synthetic aperture window apodization function with a volumetric SAFT algorithm as an expansion of the commonly used 1D apodization window in the planar SAFT algorithm [25]. In the last step, we apply a threshold to reject data in the resulting image with intensity amplitude below a certain threshold as an increased enhancement to the final image, resulting in reduced defect size error.

## 2. Improved SAFT Algorithm Model

The standard SAFT algorithm is used under different conditions for the signal processing in the NDT technique for the detection of embedded defects. The analysis of the signal can be performed in the time or frequency domain. Different setup arrangements have been proposed, such as pulse-echo or pitch-catch modes. In the time domain analysis, based on the DAS methodology, the SAFT provides a focused image of the defect inside the object with a SNR higher than that of other techniques that rely mainly on fewer measurements to detect the presence of a defect. A specific approach has to be considered to create a 3D reconstruction of a volume of interest in order to investigate a particular suspected embedded defect. In this regard, we propose the generation of multiple A-scan measurements at various points with predetermined spacing in both horizontal and vertical directions, creating a 2D scan area. Each A-scan signals comprises the superposition of the contribution of individual scatterers inside the volume of interest. Hence, each time instant is represented by a specific amplitude corresponding to the influence of scatterers that lie inside the active volume at a TOF equivalent to their distance from the exciter-receiver, respectively [22]. 

For an arbitrary excitation point, *T*, that generates an ultrasound wave propagating in the volume and an arbitrary receiver, *R*, which detects the reflected signal by the volume points, one of which is the point of interest, *P*, we can define the TOF of the signal as [17,19,23]

(1) $$TOF_{(i,j,k)P_{}}=\frac{\left|{\vec{d}}_{{}_{(i,j,k)P}}-{\vec{d}}_{{}_{(i,j,z)T}}|+|{\vec{d}}_{{}_{(i,j,k)P}}-{\vec{d}}_{{}_{(i,j,z)R}}\right|}{c}$$

where the *TOF* is the time-of-flight of the ultrasonic signal generated by the exciter *T*, scattered from the point *P* and detected by the receiver *R*; $\vec{d}$ is the displacement vector at positions *T*, *R*, and *P*, respectively; *i*, *j*, and *k* represent the indexes of the volume image points in the *X*, *Y*, and *Z* planes respectively; and *c* denotes the longitudinal speed of sound in the material of the object.

Shifting the position of the exciter/receiver results in a different *TOF* for the same particular scatterer in the active volume and a different amplitude at the corresponding time instant at the A-scan signal. The summation of all these amplitudes with respect to their TOFs (Equation (2)) will generate a focused image, $y_{f}(p)$, of that scatter with an overall amplitude corresponding to all scan points in the 2D scan area.

(2) $$y_{f}(P)=\sum_{j=1}^{N}\sum_{i=1}^{M}y_{r}(TOF_{{\left(i,j,k\right)}_{P}},i,j)$$

where $y_{r}$is a preliminary unfocused image of this particular point *P*; $y_{f}$ is the high-resolution focused image; and M*N are total scan points in horizontal and vertical directions of the scan area. We note that Equation (2) does not take into account the nature of propagating ultrasound in the solid object, which mostly contains main and side lobes at different angles and intensities.

The above-mentioned discussion referred to the standard SAFT algorithm methodology. In our approach we propose the use of the apodization function to enhance the resulting image by weighing the amplitudes resulting from the propagation of side lobes that can produce secondary echoes that influence and distort the main echoes generated by the scatterers (Equation (3)).

(3) $$y_{f}(P)=\sum_{j=1}^{N}\sum_{i=1}^{M}a(TOF_{{\left(i,j\right)}_{P}},i,j)*y_{r}(TOF_{{\left(i,j,k\right)}_{P}},i,j)$$

where $a(TOF_{{\left(i,j,k\right)}_{p}},i,j)$ is the weighing or apodization function [36]. Equation (3) represents the above idea of DAS, where the summation is applied to the delayed versions of the signals at the corresponding scan points. The apodization function is generally chosen to approach zero at the edges of the SA window edges [24].

In the SAFT analysis, if the scan is performed in a line of scan to generate a reconstruction image in a 2D plane, i.e., scan in one line and inspection of the depth of the object in the plane containing that line, we use a 1D apodization window that represents the aperture line at each point of the scatterers in the cross-section depth of the object. This approach is well-known in the literature [24,25,26,27]. We propose an improvement to this approach by extending this apodization to the 3D case. When the SAFT scan is performed in a 2D area to perform a 3D reconstruction image, the aperture of the apodization window also becomes a 2D area. To make it clearer, the dimension of the apodization window is the same as the dimension of the scan line/area, as it depends on the position of the scan sensors.

To clarify the meaning of the apodization function we first describe this concept in the commonly used case of a 1D scan line on X-axis and scatter plane XZ. Later we will expand the discussion to the general case of 2D scan area and scatterer volume. The expansion to a 2D apodization window has been previously discussed [25]. However, to date, and to our knowledge, it has not been used for SAFT analysis.

**Figure 1:**
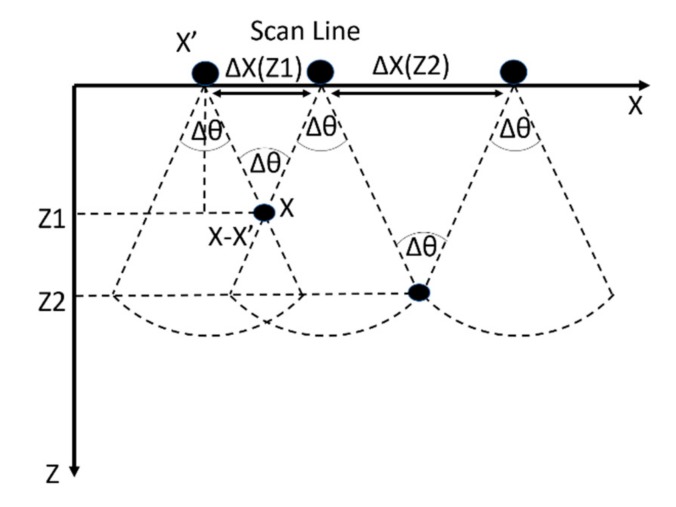
Beamwidth angle Δ*θ* for a point scatterer at depth *Z*; when the point scatterer is deeper *Z2* > *Z1*, the synthetic aperture size (Δ*X*) changes accordingly. The apodization window size should be adapted to the synthetic aperture window size.

For a 1D scan line X-axis and point scatterers in a plane XZ, the width of the apodization function should be proportional to the depth of the scatter point (Figure 1) and it is denoted by Equation (4) below [24].

(4) $$\Delta X(Z)=2Z\tan(\Delta \theta_{X}/2)$$

where ΔX(Z) is the width of the apodization window, *Z* is the depth of the point scatter, and Δθ is the angular beam width of the transducer and can be calculated at −6 dB based on Equation (5) [24,37]:

(5) $$\sin(\Delta \theta_{X}/2)=0.514*\frac{c}{fD}$$

where *f* is the central frequency and *D* is the diameter of the transducer.

The apodization function can be applied to all points falling within a certain normalized X coordinate, $\hat{X}$, and neglecting all other measurements outside this threshold (Equation (6)).

(6) $$\hat{X}=\frac{X-X\prime }{\Delta X(Z)}$$

where *X* − *X’* is the horizontal shift between the position of the scan point and scatterer.

The most commonly used apodization functions are the rectangular or Hanning function [24]. Equation (7) and Equation (8) represent the apodization functions $a(\hat{X})$ for rectangular and Hanning windows, respectively, for a 1D scan line on the X-axis [24,25].

(7) $$a{\left(\hat{X}\right)}_{rect}=\left\{\begin{array}{ll} 1, & \left|\hat{X}\right|<\alpha \\ 0, & otherwise \end{array}\right.$$

(8) $$a{\left(\hat{X}\right)}_{Hann}=\left\{\begin{array}{ll} 0.5[1+\cos(\frac{\pi }{\alpha }\hat{X})], & \left|\hat{X}\right|<\alpha \\ 0, & otherwise \end{array}\right.$$

Optimum results are obtained by fine-tuning the threshold criterion value, *α*. In order to apply the same apodization function to the entire volume with a 2D scan area, we require that the beamwidth angle become a solid angle instead of a planar angle. To make it simpler, we will have two angles $\Delta \theta_{X}\;,\Delta \theta_{Y}$ corresponding to the scan area XY instead of the scan line X. In this case, we deal with the apodization function $a(\hat{r})=a(\hat{X},\hat{Y})$ as a separable function (i.e., we can calculate the two dimensional function by considering a series of one dimensional functions) [25]. Hence, the one dimensional window functions in Equation (7) and Equation (8) can be transformed into the two dimensional window functions in Equations (9) and (10):

(9) $$a{\left(\hat{r}\right)}_{rect}=\left\{\begin{array}{ll} 1, & \left|\hat{r}\right|<\alpha \\ 0, & otherwise \end{array}\right.$$

(10) $$a{\left(\hat{r}\right)}_{Hann}=\left\{\begin{array}{ll} 0.5[1+\cos(\frac{\pi }{\alpha }\hat{r})], & \left|\hat{r}\right|<\alpha \\ 0, & otherwise \end{array}\right.$$

where $\hat{r}=\sqrt{{\hat{X}}^{2}+{\hat{Y}}^{2}}$ with the introduction of $\hat{Y}$ as the normalized Y coordinate $\hat{Y}=\frac{Y-Y\prime }{\Delta Y(Z)}$ and $\Delta Y(Z)=2Z\tan(\Delta \theta_{Y}/2)$.

With this generalized case, it is possible to apply the 2D apodization window function to the scan area of interest that covers the volume of interest.

In the derivation of the final image reconstruction using the SAFT technique, it is assumed that the ultrasonic transmitter is small enough to be considered as a point source, which is applicable in the case of LGU. Receivers also need to be regarded as point-like transducers, i.e., the smaller the size of the transducer, the smaller the numerical error in the algorithm.

An important constraint to this algorithm is the limited angle between transmitter and receiver. Typically, this should not be large to avoid diffraction and side lobes. To avoid calculation error, another important constraint is that the dimensions of the object should allow the transmitter scan area and the receiver locations to be far from the object’s boundaries to avoid their reflections. Another constraint of the SAFT algorithm is that it can work with ultrasonic signals propagating at a single velocity. Thus, composite or inhomogeneous materials that display dispersion violate the SAFT algorithm, unless the different wave velocities and diffraction are taken into account.

## 3. Experimental Configuration

**Figure 2:**
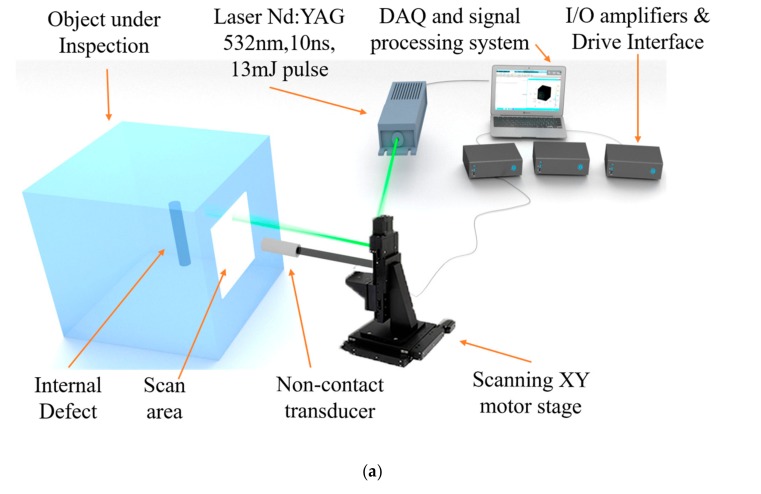

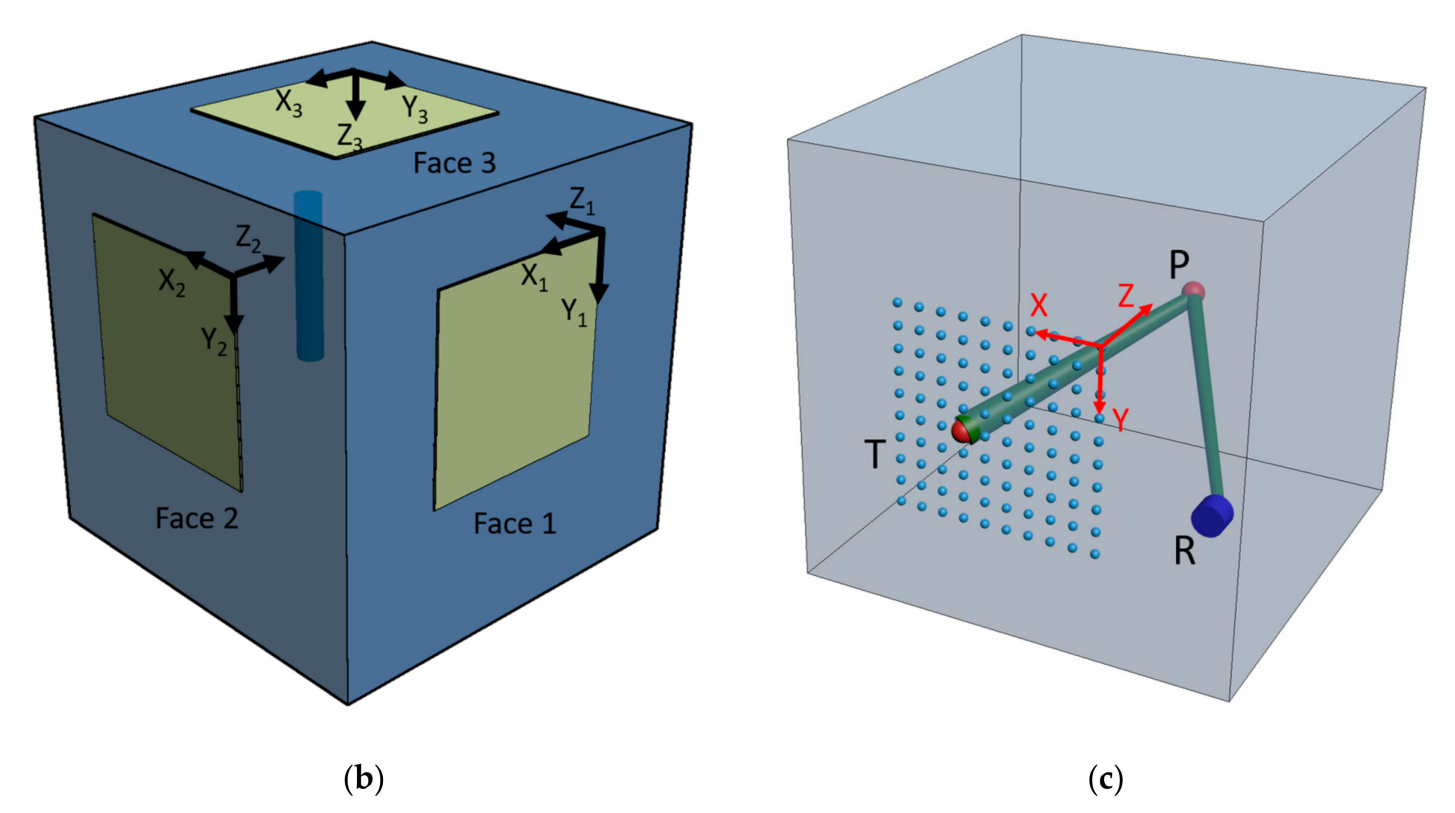
(**a**) Experimental setup used for the excitation and detection of ultrasound waves; (**b**) Schematic representation of the object under study with the three-face scan areas. Different Cartesian axes are represented at each scan face; (**c**) Representation of the 3D object under investigation using the synthetic aperture focusing technique (SAFT) algorithm with the scanning area at one of the faces with the Exciter (T), Receiver (R), and scatterer point in volume.

The schematic representation of our experimental setup and of the object under study is shown in Figure 2. The experiment was performed on a cubic sample 200 mm^3^ made of cast aluminum, with an embedded glass cylinder buried in the cube structure, representing the embedded defect. The cylinder has a 13 mm diameter and a 60 mm height, and is positioned vertically inside the cube. The defect was embedded at a depth of 100 mm from the front and side faces (face 1 and 2 in Figure 2b) and 70 mm from the top view of the cube (face 3 in Figure 2b). It should be noted that due to the fabrication process of the cast aluminum at high temperature, there is a slight tilting and bending of the glass cylinder. This results in a slight deformation and irregularity of the defect shape and location. Figure 2b also shows the scan areas of 90 × 90 mm^2^ at three faces of the cube, with scan points distributed equally in the XY planes of the three faces. Note that for simplicity each face has its own XYZ coordinate system. Figure 2c shows the schematic representation of the 3D object under investigation with the scanning area at one of the faces with the positions of the Exciter (T), Receiver (R), and scatterer point in volume, as described in Section 2.

For LGU, we used a Nd:YAG laser doubled in frequency, emitting pulses of 8 ns at a wavelength of 532 nm, with an energy per pulse of 10 mJ. The focused laser beam impacts the surface of the object under study, where it is rapidly absorbed into a shallow volume of material, creating a localized thermoelastic expansion. This expansion induces a stress wave generating broadband ultrasound waves that propagate inside the material. The scanning of the laser beam over the selected areas is performed using an XY motorized stage (OSMS CS 26-100X-M6 and OSMS26-100 Z -M6 with controllers HIT_M and HIT-S from manufacturer Optosigma) with a predefined resolution that creates 100 × 100 scan area points, equivalent to 90 × 90 mm^2^. Software based on Labview and MATLAB was prepared to control the translation stage and to define the scanning areas and number of points. A noncontact transducer (NCT2-D3, Ultran Group) with a nominal frequency of 2 MHz is fixed to the same XY motorized stage that controls the position of the laser beam spot at a fixed vertical spacing of 17 mm from the laser spot. The laser and detector move together in the scanning area. The transducer was placed at a fixed distance of 8.5 mm from the object surface. The signal collected by the sensor is sent to a preamplifier (Olympus 5662), connected to a high-performance Gage A/D card (50 MHz sampling frequency, 16-bit resolution), linked to a computer for further data processing. For each excitation point, the transducer records a voltage/time (A-scan) data set. We used longitudinal wave detection transducers considering longitudinal ultrasound wave propagation inside the aluminum material at a velocity of 6320 m/s [38,39].

## 4. Results and Discussions

We performed both excitation and detection scan over the three faces of the cube shown in Figure 2b in order to obtain detailed information of the embedded defect at different perspectives/angles, needed for the 3D reconstruction. The captured signals were first subject to the signal conditioning algorithm to obtain a higher quality signal. The algorithm involved filtering and interpolation of the measured signals to remove noise and low/high frequency components that are of no interest. Additionally, we subtracted the noise signal from the main signals to remove background effect. An averaging algorithm was also implemented to remove DC components and offsets in the measured signals. The SAFT algorithm described in Section 2 was then applied to all signals coming from the three faces scan areas. It should be noted that a 25 µs time delay is added to the TOF values extracted from Equation (3) [39]. This delay is equivalent to the velocity of ultrasound exiting the object cube at 340 m/s and travelling through the airgap of 8.5 mm towards the transducer. The 2D Hanning window apodization function was applied to the SAFT algorithm by considering the threshold criterion *α* = 0.5. This value is applied by substitution in Equation (10). Any value of $\hat{r}$ > 0.5 was rejected and =0. The calculation is applied for each specific point scatterer in the volume with respect to all exciter and receiver positions. The result is substituted in Equation (3) for each point scatterer to apply the weighting to the SAFT image. We used the same criterion as validated and investigated in [24], so that cancellation of sidelobes is achieved [24,25]. The beamwidth is frequency-dependent, and the width of the apodization function cannot match the beamwidth for all frequencies within the transducer passband. To cover the complete synthetic aperture, the apodization function must be tuned to the beamwidth by taking into account the lower edge frequency of the bandpass filter [24]. Note that a narrow apodization window increases the amplitude of the main lobe echoes, but also increases the side lobe effects and their redundant shadows, while a wider apodization window reduces sidelobe effects but at same time distorts the focusing of the main lobe and reduces the focusing amplitude [24,25]. The selection of the optimum value of the threshold is subject to a tuning process. We applied fine-tuning of the value of threshold value between 0.3 and 0.7. The best results were obtained at a value of 0.5 similar to [24,25]. Fine-tuning is applied to optimally select the 25-µs time delay corresponding to the airgap effect. 

**Figure 3:**
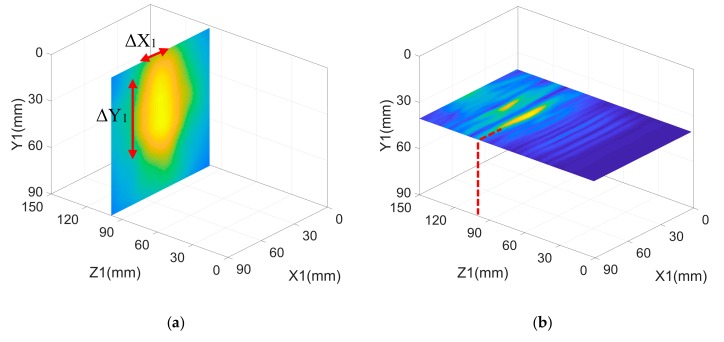

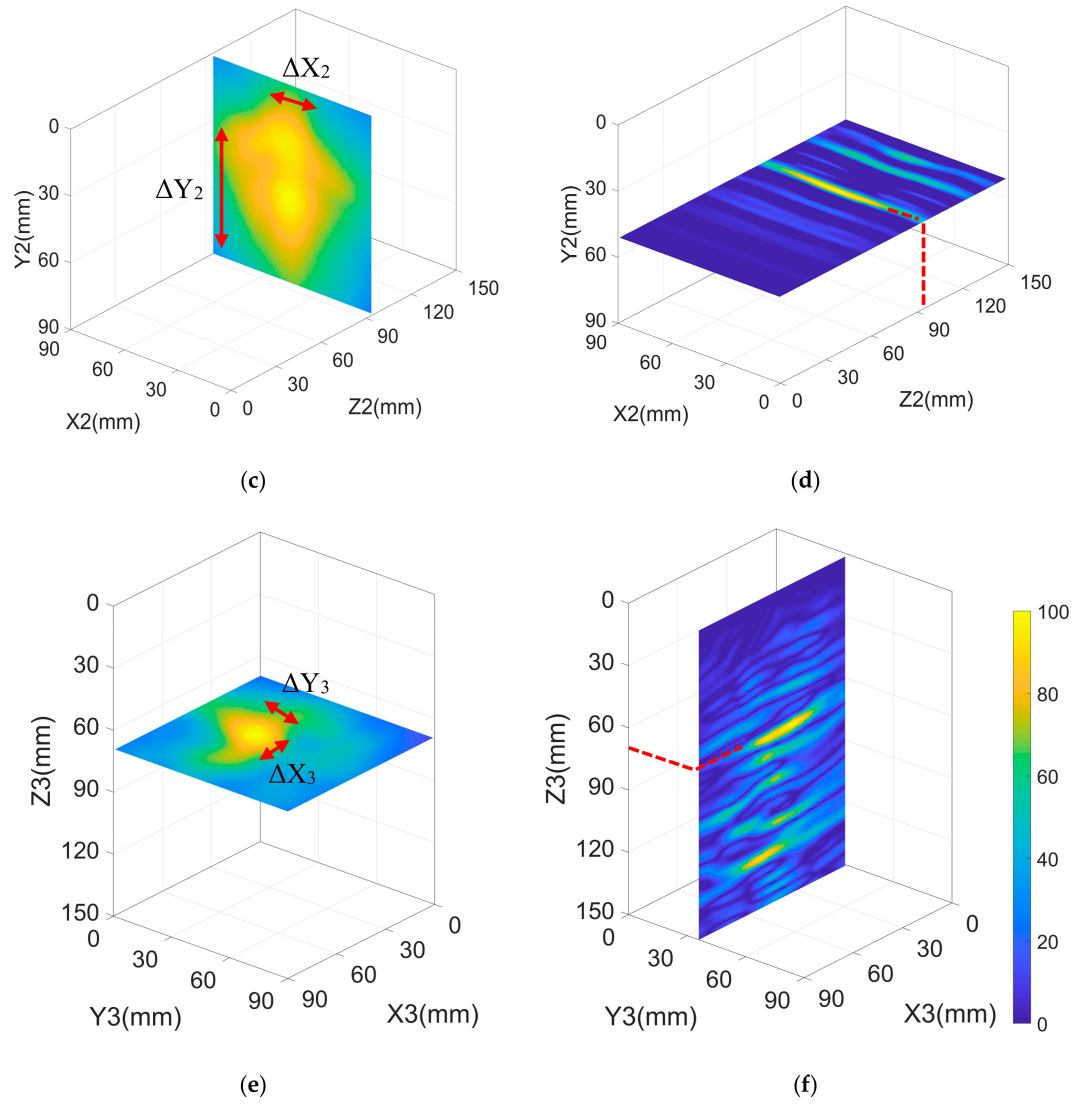
The SAFT algorithm results of the three-face experiment: (**a**) X_1_Y_1_ plane slice for face 1 at Z_1_ = 99 mm; (**b**) X_1_Z_1_ plane slice for face 1 at Y_1_ = 40 mm; (**c**) X_2_Y_2_ plane slice for face 2 at Z_2_ = 95 mm; (**d**) X_2_Z_2_ plane slice for face 2 at Y_2_ = 51 mm; and (**e**) X_3_Y_3_ plane slice for face 3 at Z_3_ = 70 mm f) X_3_Z_3_ plane slice for face 3 at Y_3_ = 36 mm. (color map at bottom right applies to all subfigures).

The results of our full noncontact experiment for each scan area are shown in Figure 3. The defect is located and represented for each scan face at positions corresponding to the larger signal amplitude shown in the figures. The Cartesian axes X, Y, and Z are chosen for each face to cover only the respective scanned area and have their origin at the top right corner, with XY being the scanned area plane and the Z axis is perpendicular to this area. The axes for each face are denoted by X_1_, Y_1_, and Z_1_ (face 1); X_2_, Y_2_, and Z_2_ (face 2); and X_3_, Y_3_, and Z_3_ (face 3), respectively. The results below for the three faces are represented at XY and XZ cross sections respectively. The color map at bottom right of Figure 3 represents the amplitude percentage and applies to all subfigures.

For face 1, we have the front view of the defect in the X_1_Y_1_ (Figure 3a) and X_1_Z_1_ (Figure 3b) planes centered at X_1_ = 41 mm, Y_1_ = 41 mm, and at Z_1_ = 99 mm. The reconstructed defect dimensions are ΔX_1_ = 18 mm and ΔY_1_ = 55 mm, which correspond to a size error with respect to the original defect size of ΔX_1,error_ = 38% and ΔY_1,error_ = 8%, and a positioning error of less than 1% in X_1_,Y_1_, and Z_1_ planes. The positioning error refers to the difference between the actual position of the defect’s center in X_1_,Y_1_, and Z_1_ planes and the detected ones. With regard to face 2, we see the side view of the defect in the X_2_Y_2_ and X_2_Z_2_ planes centered at X_2_ = 49 mm, Y_2_ = 45 mm, and Z_2_ = 95 mm. The detected defect dimensions are ΔX_2_ = 19 mm and ΔY_2_ = 58 mm. This corresponds to a size error with respect to the original defect size of ΔX_2,error_ = 46%, ΔY_2,error_ = 3%, and a positioning error less than 5% in X_2_,Y_2_, and Z_2_ planes. Face 3 shows the top view of the defect in the X_3_Y_3_ and X_3_Z_3_ planes centered at X_3_ = 42 mm, Y_3_ = 34 mm, and at Z_3_ = 70 mm; the detected defect dimensions are ΔX_3_ = 16 mm, ΔY_3_ = 16 mm, which corresponds to a size error with respect to original defect size of ΔX_3,error_ = 23%, ΔY_3,error_ = 23%, and a positioning error less than 1% in X_3_, Y_3_, and Z_3_ planes. We emphasize that the ΔX_error_ magnitude in either face is related to the transducer’s size, 13 mm in our case. It is not possible for the sensor to accurately measure the size of defects below or equal to the transducer’s size. Indeed, the size error ΔY_error_ is small because the defect size in the Y dimension is much larger than the transducer’ size. Therefore, although the resulting size and positioning estimates obtained from each face show good global performance, the errors can be drastically reduced by using smaller size transducers.

**Figure 4:**
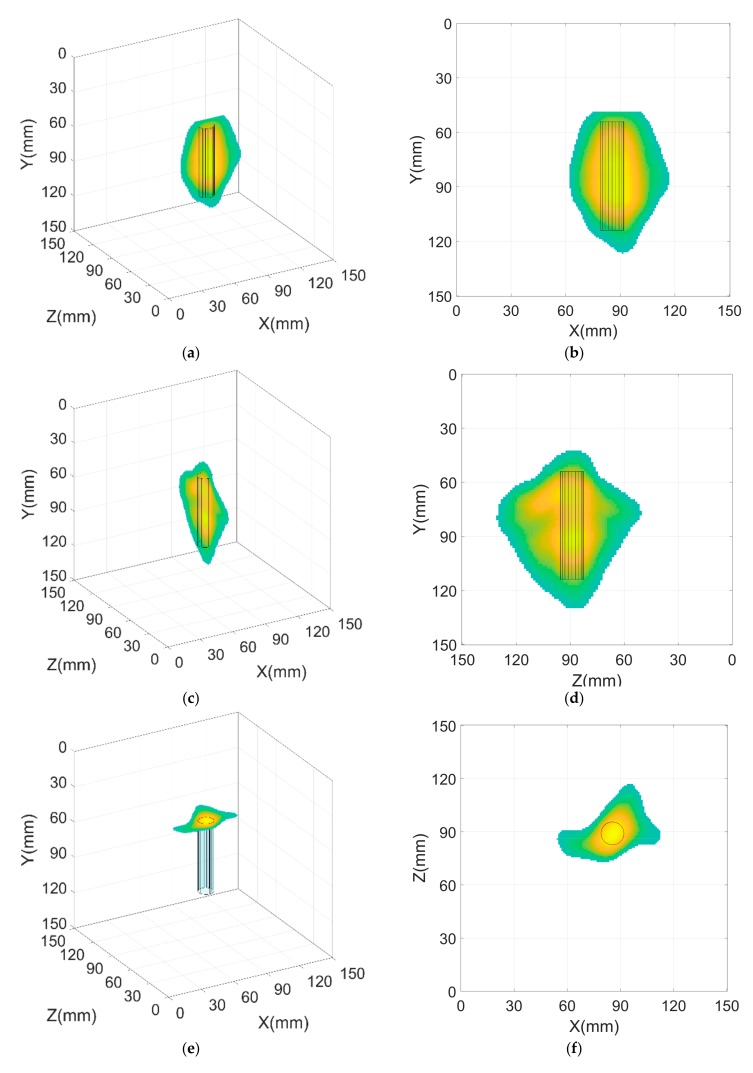

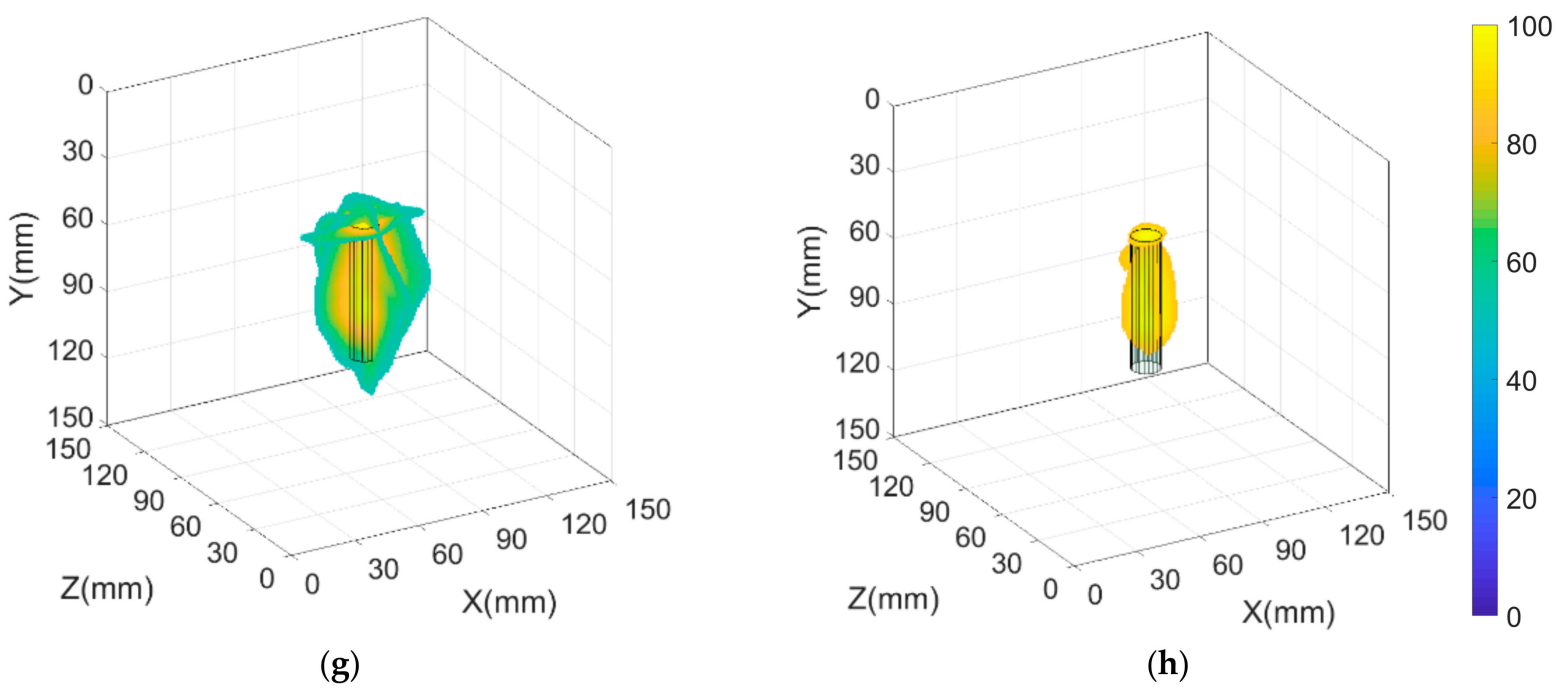
3D reconstruction of the defect by combining the resulting SAFT images from the three faces and superimposing the actual cylindrical shape of the defect. (**a**) Isometric view resulting from face 1 (front view/X_1_Y_1_) scan area inspection by applying a reject threshold for data with an intensity below 65%; (**b**) Front view resulting from face 1 scan area inspection by applying a reject threshold for data with intensity below 65%; (**c**) Isometric view resulting from face 2 (Side view/X_2_Y_2_) scan area inspection by applying a reject threshold for data with an intensity below 65%; (**d**) Side view resulting from face 2 scan area inspection by applying a reject threshold for data with an intensity below 65%; (**e**) Isometric view resulting from face 3 (Top view/X_3_Y_3_) scan area inspection by applying a reject threshold for data with an intensity below 65%; (**f**) Top view resulting from face 3 scan area inspection by applying a reject threshold for data with an intensity below 65%; (**g**) Isometric view resulting from the 3d reconstruction by applying a reject threshold for data with an intensity below 65%; (**h**) isometric view by applying a stricter reject threshold for data with an intensity below 85%. (Color map at bottom right applies to all subfigures.)

Combining all the three faces views and representing them at the scale of the cube, we may generate the 3D reconstruction of the defect as shown in Figure 4. Here, a unique universal Cartesian axis reference XYZ is used for all the three faces with the origin in the corner of the cube object itself. The relative displacement between the scan faces is considered when superimposing the scan faces all together on the 3D reconstruction. We applied a thresholding filter to reject all data in Figure 3 below a certain amplitude to keep only the high intensity data to represent the defect position and shape. In our case, we selected two thresholds—65% and 85%—as the limits of the rejection of data. That was chosen after visualizing the unfiltered reconstructed images (Figure 3). We found that the points with high intensity begin around color map of 75%, and so we decided to provide a ±10% margin below and above these limits to make our upper and lower thresholds 65% and 85%, respectively. The choice of the intensity reject threshold is a user-decision as in any image processing filter. Therefore, some care should be exercised when applying this filter in order to obtain good results without affecting image fidelity and quality. Applying these thresholds with proper fine-tuning of the limits also helps reduce the size error, improving the accuracy of the algorithm. Figure 4a shows the Isometric view resulting from face 1 (Front view/X_1_Y_1_) scan area inspection by applying a reject threshold for data with intensity below 65%, while Figure 4b shows the front view resulting from face 1 scan area inspection by applying a reject threshold for data with intensity below 65%. Figure 4c,d shows the isometric view and side view representations respectively for face 2 with the same threshold condition. Figure 4e,f shows the isometric view and top view representations respectively for face3. Figure 4g shows the isometric view including the intersection between reconstruction results from the three faces with a filtering threshold of intensity reject for values less than 65%. Figure 4h shows the isometric view including the intersection between reconstruction results from the three faces with a more restrictive threshold of intensity to reject data with intensity below 85%. We superimpose the true cylindrical defect shape on the reconstructed defect images to see how close the detected information is, with respect to true information. The color map at bottom right of Figure 4 represents the amplitude percentage and applies to all subfigures.

It is clear that the detected defect size and position match the true defect size and position. Increasing the number of scanned faces results in higher resolution reconstruction of the defect from all 360° angles. The reconstructed planes in our case show a high contrast between the points of high intensity (corresponding to the presence of the defect) and the points of low intensity (no defect). The use of the apodization function helped increase this contrast and eliminated the effect of reflections from side lobes or signals at large angles. The size error is enhanced for the case of a threshold of 65% to be less than 10% for the three faces in the horizontal dimension. In the case of 85% threshold, the size error is reduced to less than 5% in the horizontal dimension. In the vertical dimension, the size error is not affected by a significant change, with the same error as in the case of no threshold. In our case, the optimum threshold after the fine-tuning was 85%, which strongly reduced the error in the horizontal dimension.

The 3D reconstruction of the defect in Figure 4 clearly shows that the improved SAFT method used in this work has powerful advantages in visualizing the defect in the 3D isometric view. The projections of the SAFT planes make it easier to distinguish the location of the defect in a 3D manner instead of just getting two dimensional results. The SAFT algorithms using a hybrid technique allows scanning over large objects without significant loss of information. In fact, we obtain better localized quality image of the defect with larger objects compared with smaller ones due to the aforementioned boundary conditions. 

The precision of the results obtained here can be further improved using a noncontact transducer having smaller size, adapted to the horizontal size of the defect. The resolution would be enhanced and calculated accurately with less error. Another significant qualitative enhancement that results from using the noncontact transducer is a cleaner signal with a higher intensity contrast due to having the receiving transducer closer to the exciter. However, there are also some drawbacks. For example, the noncontact transducer must be placed very close to the exciter because otherwise the signal can be overly attenuated. Also, the airgap can affect the quality of the signal. This can be remedied by using a high-power pulsed laser exciter and high-gain preamplifier. Other critical factors related the experimental setup and the signal processing algorithm need to be considered. For instance, the SAFT algorithm is having limitations considering the angle between the exciter and receiver that should be small to avoid diffraction and side-lobe effects. This is already overcome in our case since our experimental setup was configured to place the exciting Laser and the receiving transducer adjacent to each other. Another important factor is the correct choice of the threshold criterion for apodization algorithm to maintain the lateral resolution of the main lobe and get rid of the effect of the side-lobes. Finally, the selection of the amplitude threshold, to the resulting SAFT 3D reconstructed image, is important to be chosen carefully to improve the size and position error, while maintaining the correct dimensions of the defect.

An important advantage of the proposed method is that there is no need to use a healthy reference sample to compare with the resulting reconstruction image of the unhealthy sample to detect the presence of the defects. However, in the case of an object that has no symmetrical dimensions or has a composite structure of different materials, it would help to use the healthy reference sample, to avoid misrepresenting the internal reflections from object’s boundaries. 

## 5. Conclusions

We have demonstrated that using a hybrid method composed by LGU as an exciter, and noncontact transducer as a detector, we can design a fully noncontact configuration for NDT inspection and 3D reconstruction of a defect embedded in a metallic object. The use of LGU allows remote excitation of large scan areas with higher power levels, not achievable with conventional transducers, which helps with sample penetration and embedded defect detection. On the other hand, noncontact transducers have the advantage of having a large number of points scan and proximity to the exciter, which allows the detection of more details of the reflected ultrasound waves. The combination of signals coming from three orthogonal scanning areas of the object detects the defect’s presence from all perspectives/angles. An improved SAFT algorithm is implemented to localize the defect position with a signal to noise ratio, taking into consideration the limitations of structural dimensions. The SAFT algorithm provides more information about the exact location of the defect using 3D reconstruction imaging. The use of the 2D apodization technique helped enhance the SNR ratio and reject the effect of side lobes excited along with the main ultrasound lobes, as well as the effect of wide-angle reflections. The hybrid, fully noncontact techniques presented here provide a strong alternative to conventional NDT techniques, with higher flexibility, higher resolution and powerful detection of embedded defects.

## References

[1] Her S.C., Lin S.T. (2014). Non-destructive evaluation of depth of surface cracks using ultrasonic frequency analysis. Sensors.

[2] Rose J.L. (2004). Ultrasonic Waves in Solid Media.

[3] Ostachowicz W., Kudela P., Krawczuk M., Zak A. (2012). Guided Waves in Structures for SHM: The Time-Domain Spectral Element Method.

[4] Naugolnykh K.A., Ostrovsky L. (1998). Nonlinear Wave Processes in Acoustics.

[5] Mi B., Michaels J.E., Michaels T.E. (2006). An ultrasonic method for dynamic monitoring of fatigue crack initiation and growth. J. Acoust. Soc. Am..

[6] Cheng Y., Deng Y., Cao J., Xiong X., Bai L., Li Z. (2013). Multi-wave and hybrid imaging techniques: A new direction for nondestructive testing and structural health monitoring. Sensors.

[7] Delrue S., van Den Abeele K., Blomme E., Deveugele J., Lust P., Matar O.B. (2010). Two-dimensional simulation of the single-sided air-coupled ultrasonic pitch-catch technique for non-destructive testing. Ultrasonics.

[8] Delrue S., Tabatabaeipour M., Hettler J., van Den Abeele K. (2016). Applying a nonlinear, pitch-catch, ultrasonic technique for the detection of kissing bonds in friction stir welds. Ultrasonics.

[9] Bai Z., Chen S., Xiao Q., Jia L., Zhao Y., Zeng Z. (2018). Compressive sensing of phased array ultrasonic signal in defect detection: Simulation study and experimental verification. Struct. Heal. Monit..

[10] Ciampa F., Mankar A., Marini A. (2017). Phononic crystal waveguide transducers for nonlinear elastic wave sensing. Sci. Rep..

[11] Miniaci M., Gliozzi A.S., Morvan B., Krushynska A., Bosia F., Scalerandi M., Pugno N.M. (2017). Proof of concept for an ultrasensitive technique to detect and localize sources of elastic nonlinearity using phononic crystals. Phys. Rev. Lett..

[12] Tiwari K.A., Raisutis R., Tumsys O., Ostreika A., Jankauskas K., Jakutavicius J. (2019). Defect estimation in non-destructive testing of composites by ultrasonic guided waves and image processing. Electronics.

[13] Le M., Kim J., Kim S., Lee J. (2016). Nondestructive testing of pitting corrosion cracks in rivet of multilayer structures. Int. J. Precis. Eng. Manuf..

[14] Praveen A., Nikhilesh, Vijayarekha K., Manjula K., Venkatraman B. (2012). Wavelet analysis and de-noising of signal. Res. J. Appl. Sci. Eng. Technol..

[15] Selim H., Prieto M.D., Trull J., Romeral L., Cojocaru C. (2019). Laser ultrasound inspection based on wavelet transform and data clustering for defect estimation in metallic samples. Sensors.

[16] Prada C., Kerbrat E., Cassereau D., Fink M. (2002). Time reversal techniques in ultrasonic nondestructive testing of scattering media. Inverse Probl..

[17] Spies M., Rieder H., Dillhöfer A., Schmitz V., Müller W. (2012). Synthetic aperture focusing and time-of-flight diffraction ultrasonic imaging—Past and present. J. Nondestruct. Eval..

[18] Tiwari K.A., Raisutis R., Samaitis V. (2017). Hybrid signal processing technique to improve the defect estimation in ultrasonic non-destructive testing of composite structures. Sensors.

[19] Boonsang S., Zainal J., Dewhurst R.J. (2004). Synthetic aperture focusing techniques in time and frequency domains for photoacoustic imaging. Insight Non-Destructive Test. Cond. Monit..

[20] Guarneri G.A., Pipa D.R., Neves F.J., de Arruda L.V.R., Zibetti M.V.W. (2015). A sparse reconstruction algorithm for ultrasonic images in nondestructive testing. Sensors.

[21] Gómez M.J., Castejón C., García-Prada J.C. (2016). Review of recent advances in the application of the wavelet transform to diagnose cracked rotors. Algorithms.

[22] Selim H., Delgado M., Trull J., Picó R., Cojocaru C. (2018). Material defect reconstruction by non-destructive testing with laser induced ultrasonics. J. Phys. Conf. Ser..

[23] Stepinski T., Lingvall F. Synthetic aperture focusing techniques for ultrasonic imaging of solid objects. Proceedings of the 8th European Conference on Synthetic Aperture Radar.

[24] Martin H.S. (2012). Synthetic Aperture Ultrasound Imaging with Application to Interior Pipe Inspection. Ph.D. Thesis.

[25] Widada W. (1979). Two Dimensional Window Functions. Master’s Thesis.

[26] Cobbold R.S.C. (2013). Corrections to Foundations of Biomedical Ultrasound.

[27] Szabo T.L. (2004). Diagnostic Ultrasound Imaging: Inside Out.

[28] De Marchi L., Marzani A., Miniaci M. (2013). A dispersion compensation procedure to extend pulse-echo defects location to irregular waveguides. NDT E Int..

[29] Krohn N., Pfleiderer K., Stoessel R., Solodov I., Busse G. (2004). Nonlinear acoustic imaging: Fundamentals, methodology, and NDE-applications. Acoustical Imaging.

[30] Ulrich T.J., Johnson P.A., Sutin A. (2006). Imaging nonlinear scatterers applying the time reversal mirror. J. Acoust. Soc. Am..

[31] Miniaci M., Mazzotti M., Radzieński M., Kudela P., Kherraz N., Bosia F., Pugno N.M., Ostachowicz W. (2019). Application of a laser-based time reversal algorithm for impact localization in a stiffened aluminum plate. Front. Mater..

[32] Kreis T. (2016). Application of digital holography for nondestructive testing and metrology: A review. IEEE Trans. Ind. Inf..

[33] Zhang K., Zhou Z., Zhou J. (2015). Application of laser ultrasonic method for on-line monitoring of friction stir spot welding process. Appl. Opt..

[34] Streza M., Dadarlat D., Fedala Y., Longuemart S. (2013). Depth estimation of surface cracks on metallic components by means of lock-in thermography. Rev. Sci. Instrum..

[35] Jen C.K., Wu K.T., Kobayashi M., Blouin A. NDE using laser generated ultrasound and ultrasonic transducer receivers. Proceedings of the 2008 IEEE Ultrasonics Symposium.

[36] Jensen J.A., Nikolov S.I., Gammelmark K.L., Pedersen M.H. (2006). Synthetic aperture ultrasound imaging. Ultrasonics.

[37] Ultrasonic Transducers. Vol. Pana_UT_EN. http://www.epsilon-ndt.com/upload/file/problar-ve-aksesuarlar-.pdf.

[38] Cong S., Zhang W.W., Zhang J.Y., Gang T. (2017). Analysis on Ultrasonic TOFD Imaging testing for ultra-thick-walled ebw joint of aluminum alloy. Proc. Eng..

[39] Wang X.G., Wu W.L., Huang Z.C., Chang J.J., Wu N.X. (2018). Research on the transmission characteristics of air-coupled ultrasound in double-layered bonded structures. Materials.

